# Somatic Mutation of *NLRP* Genes in Gastric and Colonic Cancers

**DOI:** 10.3389/pore.2021.607385

**Published:** 2021-04-16

**Authors:** Seong Won Moon, Hyun Ji Son, Ha Yoon Mo, Nam Jin Yoo, Sug Hyung Lee

**Affiliations:** ^1^ Department of Pathology, College of Medicine, The Catholic University of Korea, Seoul, South Korea; ^2^ Department of Cancer Research Institute, College of Medicine, The Catholic University of Korea, Seoul, South Korea

**Keywords:** NLRP, Somatic mutation, loss of expression, cancer, colon cancer

## Abstract

Nucleotide-binding and leucine-rich repeat protein (NLRP) genes are involved in inflammasome formation that plays a role in inflammation/host defense and cell death. Both cell death and inflammation are crucial for cancer development, but the roles of NLRPs in cancer are partially known. In this study, we analyzed mononucleotide repeats in coding sequences of *NLRP1, NLRP2, NLRP4* and *NLRP9,* and found 1, 1, 1 and 8 frameshift mutation (s) in gastric (GC) and colonic cancers (CRC), respectively. Five of the 32 high microsatellite instability (MSI-H) GCs (15.5%) and 6 of 113 MSI-H CRCs (5.5%) exhibited the frameshift mutations. There was no *NLRP* frameshift mutations in microsatellite stable (MSS) GCs and CRCs. We also discovered that 2 of 16 CRCs (12.5%) harbored intratumoral heterogeneity (ITH) of the *NLRP9* frameshift mutations in one or more areas. In both GC and CRC with MSI-H, NLRP9 expression in *NLRP9*-mutated cases was significantly lower than that in *NLRP9*-non-mutated cases. Our data indicate that *NLRP9* is altered at multiple levels (frameshift mutation, mutational ITH and loss of expression), which together could contribute to pathogenesis of MSI-H GC and CRC.

## Introduction

Nucleotide-binding and leucine-rich repeat proteins (NLRPs), also known as NALPs, are crucial mediators in inflammation and host defense [1–3]. Currently, there are 10 known human *NLRP* genes (*NLRP1*-6*, 9, 10, 12, 14*). They, in common, possess pyrin, NACHT and leucine-rich repeat domains and are crucial for aggregating other proteins that form the inflammasome [1–3]. Of the 10 *NLRP* genes, gene functions of *NLRP1, 3* and *5* have been known well [4–6]. NLRP3 inflammasome formation enables activation of caspase-1 and subsequent interleukin-1β and interleukin-18 activation, which could develop cell death and inflammation [7]. Both cell death and inflammation are crucial for cancer development, but the roles of NLRPs in cancer development remain controversial due to the diverse cancer-related findings [1, 5]. For example, recent studies identified NLRPs that modulated the mucosal immune response during inflammatory bowel disease-associated tumorigenesis [8, 9]. NLRP1 inflammasome attenuates colitis and colitis-associated tumorigenesis [9]. In skin squamous cell carcinoma, NLRP1 inflammasome pathway is silenced [10]. Also, NLRP2 inhibits cell proliferation and tumor growth of human glioblastoma [11]. NLRC3, a putative NLR, has anti-cancerous functions [12]. These data suggest tumor suppressor gene (TSG) functions of NLRPs in cancer development. By contrast, tumor-promoting (oncogenic) functions of NLRPs have been discovered in many cancers as well. NLRP1 overexpression is correlated with the tumorigenesis and proliferation of human breast cancer [13]. In melanoma, NLRP1 promotes tumor growth by enhancing inflammasome activation and suppressing apoptosis [14]. Activation of NLRP3 inflammasome promotes inflammation-induced tumor growth and metastasis in many cancers [2]. These data may indicate that NLRPs are involved in cancer pathogenesis, but their cancer-related alterations vary depending on cancer types.

DNA mismatch repair (MMR) is a cellular mechanism for correcting erroneous bases by MMR-specific proteins, alterations of which would result in microsatellite instability (MSI) and mutator phenotypes [15]. The mutator phenotype is characterized by mutation aCRCumulation in repetitive DNA sequences (frequently mono- or dinucleotide repeats). In coding DNA sequences, the MSI produces frameshift mutations within the affected proteins that would truncate protein synthesis [16]. Gastric (GC), colonic (CRC) and endometrial cancers are the most common cancers with high MSI (MSI-H) phenotype [17]. It is believed that MSI is random, but there is evidence suggesting that MSI targets include a growing list of cancer genes such as *TGF-β1* gene and *BAX* gene [18, 19]. There are nucleotide repeats in coding DNA sequences of *NLRP1, 2, 4* and *9*, which could be altered in MSI cancers. In the present study, we detected frequent *NLRP1, 2, 4* and *9* frameshift mutations in GC and CRC with MSI-H.

## Materials and Methods

### Cancer Tissues

In the present study, formalin-fixed and paraffin embedded (FFPE) tissues of 235 cancers from Korean patients (77 GCs and 158 CRCs) were used (Table 1). Briefly, they consisted of 32 GCs with MSI-H, 45 GCs with microsatellite stable (MSS), 113 CRCs with MSI-H and 45 CRCs with MSS. For the evaluation of the MSI status of each cancer, we adopted five mononucleotide repeats (BAT25, BAT26, NR-21, NR-24 and MONO-27) that were known to be frequently mutated in MSI-H cancers [20]. Malignant cells and normal cells were separately collected from hematoxylin-eosin slides using a 30G1/2 hypodermic needle by microdissection as described in earlier studies [21, 22]. DNA extraction was performed by a modified single-step DNA extraction method using proteinase K [21, 22]. Research approval was obtained from the institutional review board of Catholic University of Korea. All FFPE samples were made anonymous and waived the need for written informed consent.

**TABLE 1:** Summary of pathologic features of gastric and colon cancers.

Feature	MSI-H	MSS
Gastric carcinomas		
Total cases	32	45
TNM stage		
I	11	15
II	13	18
III	7	11
IV	1	1
Colon carcinomas		
Total cases	113	45
TNM stage		
I	20	6
II	50	20
III	37	16
IV	6	3
Location		
Cecum	33	0
Ascending colon	58	3
Transverse colon	15	2
Descending & sigmoid colon	6	17
Rectum	1	23

TNM: tumor, lymph node, metastasis, MSI-H: high microsatellite instability, MSS: stable microsatellite instability.

TNM stage is defined by AJCC 8th edition.

### Mutational Analysis

There is one A7 (exon 4; primers 5′-AAG​CTC​AGC​CAT​TGG​GAC​C-3′, 5′- AAG​GTG​GAG​ATG​ATG​GCC​C-3′) in *NLRP1* gene, one A7 (exon 13; primers 5′-TTT​CTT​CCC​CCA​TTG​TAC​CCC-3′, 5′- TCT​GCC​CAG​GGA​TGA​TGT​TTC-3′) in *NLRP2* gene, one T7 (exon 2; primers 5′- TCA​CCC​AGC​TGT​GAG​ATG​TG-3′, 5′- TCT​TGG​GAC​AGT​TGG​AAG​CC-3′) in *NLRP4* gene, and one A7 (exon 2; primers 5′-TGA​GCG​ATG​ATT​GGA​GGC​AG-3′, 5′-GAG​TTT​TGG​ATG​CCG​CAA​CA-3′) and one T8 (exon 1; primers 5′- CTTTTC CCT​CTG​GAG​ACA​CCT​C-3′, 5′-TTC​TCC​AAA​GGT​TGT​TTG​AGG​A-3′) in *NLRP9* gene. Genomic DNA from the microdissected cells was amplified by polymerase chain reaction (PCR) using the primer pairs. Radioisotope ([^32^P]dCTP) was incorporated into the PCR products for detection by autoradiogram. For the screening of the mutations, aberrant gel motility in single strand conformation polymorphism (SSCP) was used (FMC Mutation Detection Enhancement system; Intermountain Scientific, Kaysville, UT, United States) [21, 22]. Cancer DNA with mobility shifts in the SSCP was subsequently sequenced by Sanger DNA sequencing of both forward and reverse strands to confirm the mutated sequences (3730 DNA Analyzer, Applied Biosystem, Carlsbad, CA, United States). We also analyzed intratumoral heterogeneity (ITH) of the *NLRP9* mutations, which could be altered in MSI-H cases. For this, 16 MSI-H CRCs with 4–7 different areas per CRC were studied by PCR-SSCP and Sanger sequencing as described above.

### Expressional Analysis

Since frameshift mutations of genes in MSI-H frequently accompany expressional alteration of the affected proteins [21], we analyzed the NLRP9 protein expression status in the GCs and CRCs by immunohistochemistry using anti-NLRP9 antibody (catalogue number HPA042623, Atlas Antibodies, Stockholm, Sweden; dilution 1/50). The immunohistochemistry procedures have been described in our earlier studies [21]. Briefly, sections from FFPE GC and CRC tissues were studied using ImmPRESS System (Vector Laboratories, Burlingame, CA, United States). After deparaffinization, heat-induced epitope retrieval was conducted by immersing the slides in Coplin jars filled with 10 mmol/L citrate buffer (pH 6.0) and boiling the buffer for 30 min in a pressure cooker (Nordic Ware, Minneapolis, MN) inside a microwave oven at 700 W; the jars were then cooled for 20 min. We used diaminobenzidine (brown) as chromogen for the immunohistochemistry reactions and counterstained with hematoxylin (blue). The staining intensity was graded as follows: 0, negative; 1+, weak staining in cytosol or nucleus; 2+, moderate; and 3+, intense. The extent was graded as follows: 0, 0–5% positivity of cells; 1, 6–19%; 2, 20–49%; 3, >50%. The intensity and the extent were multiplied for the immunohistochemistry score (IS), which consisted of IS 0–2 as −, 3 or 4 as + and 6 or 9 as ++. Negative control of the immunostaining was the replacement of primary antibody with the blocking reagent.

## Results

### NLRP Gene Mutations

**FIGURE 1:**
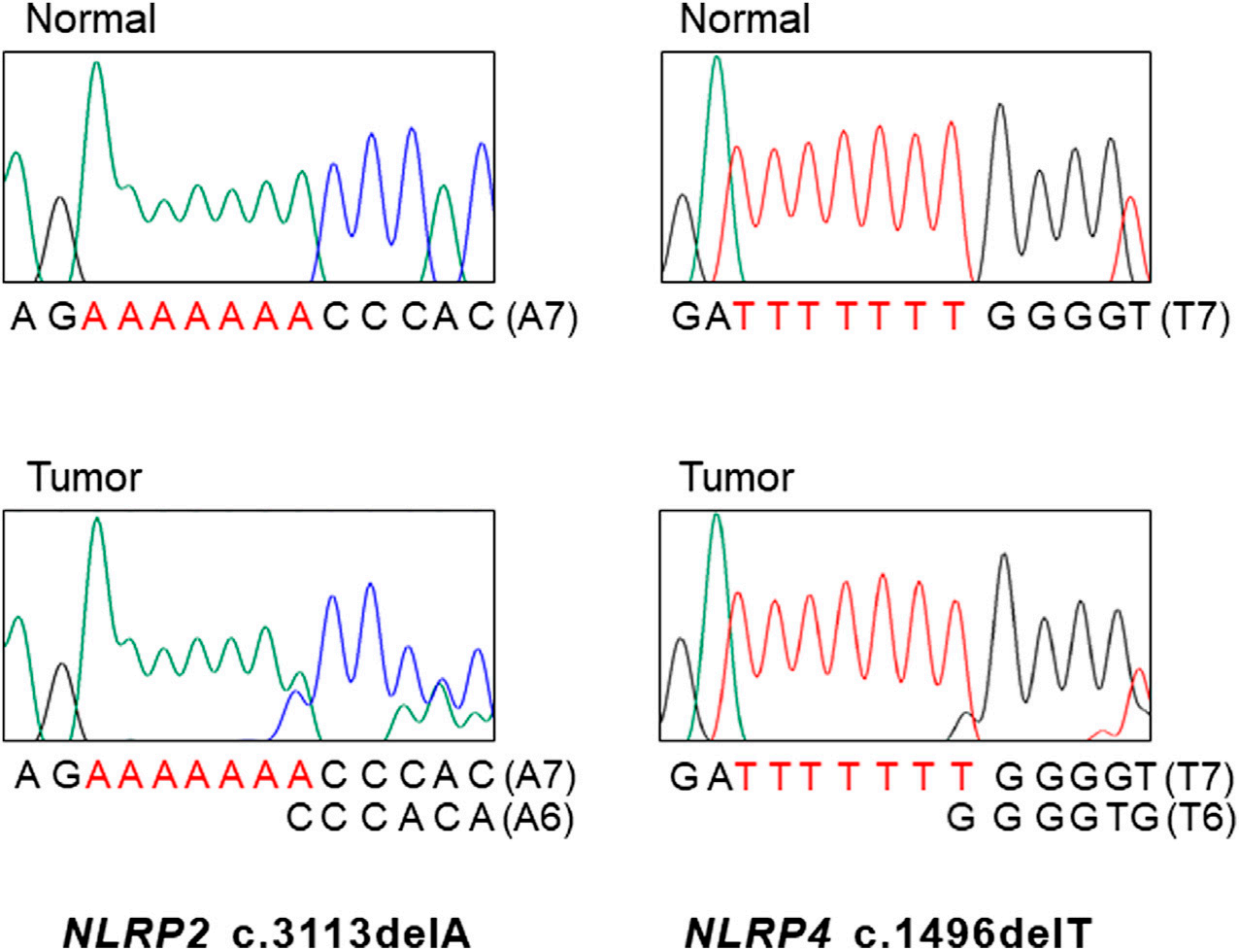
DNA sequencings *NLRP2* and *NLRP4* mutations in gastric and colon cancers. DNA sequencing analyses of the A7 repeat **(left)** of *NLRP2* and the T7 repeat **(right)** of *NLRP4* from normal **(upper)** and tumor tissues **(lower)**. Sanger DNA sequencing analyses reveal heterozygous deletions of a base within the repeats in the tumor tissues as compared to normal tissues.

**FIGURE 2:**
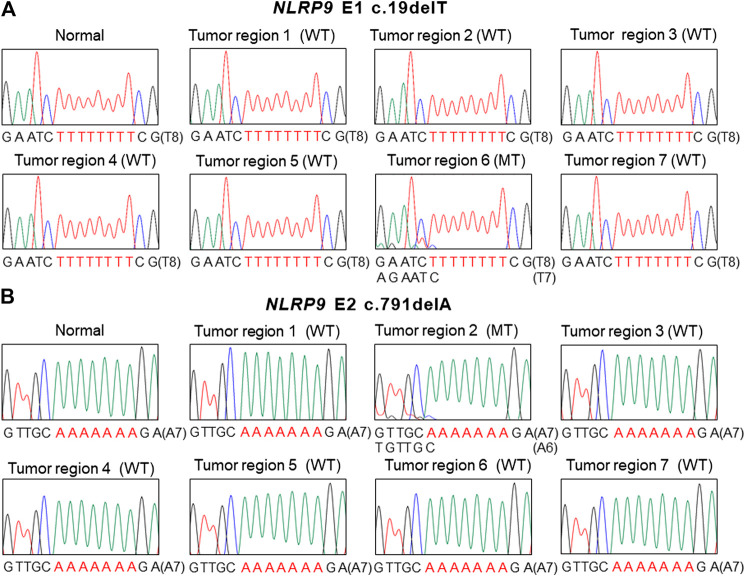
Intratumoral heterogeneity of *NLRP9* frameshift mutation in colon cancer. **(A)** Sanger DNA sequencing shows *NLRP9* c.19delT mutation (MT) in one region (6) and wild-type (WT) in the other 6 regions (1, 2, 3, 4, 5, 7). **(B**. Sanger DNA sequencing shows *NLRP9* c.791delA mutation (MT) in one region (2) and wild-type (WT) in the other 6 regions (1, 3, 4, 5, 6, 7).

The PCR-SSCP for the mononucleotide repeats in *NLRP1, 2, 4* and *9* showed aberrant migrating bands in 5 GCs and 6 CRCs, which were subsequently confirmed as frameshift mutations (deletion or duplication mutation of one base within the nucleotide repeats) by DNA sequencing (Table 2 and Figure 1). Eight, one, one and one mutation (s) were discovered in *NLRP9*, *NLRP1, NLRP2,* and *NLRP4,* respectively (Table 2)*.* Of note, all of the cancers with the *NLRP* frameshift mutations were MSI-H cases, but was none in the MSS cases (significant difference, *p* = 0.004). These cancers harbored one *NLRP* frameshift mutation in each cancer without harboring any other mutation among these genes. All the mutations were interpreted somatic as there was no such mutations in matched normal tissues. The DNA sequencing exhibited both wild-type and mutation sequences, indicating they were heterozygous mutations (Figures 1, 2). Five of the 32 MSI-H GCs (15.5%) and 6 of 113 MSI-H CRCs (5.5%) exhibited the frameshift mutations with no significant organ difference (*p* > 0.05). There was no significant difference of 5-years survival between patients with and without the mutations (*p* > 0.05).

**TABLE 2:** *NLRP* mutations in gastric and colon cancers.

Gene	Wild type	Mutation	MSI status of the mutation cases (n)	Incidence in MSI-H cancers (%)	Nucleotide change (predicted amino acid change)
*NLRP9*	T8	T9	MSI-H (1)	Gastric: 1/32 (3.1)	c.19dupT (p.Ser7PhefsX21)
		T7	MSI-H (5)	Gastric: 2/32 (6.3)	c.19delT (p.Ser7ArgfsX9)
			Colon: 3/113 (2.7)		
	A7	A6	MSI-H (2)	Gastric: 1/32 (3.1)	c.791delA (p.Lys264ArgfsX12)
				Colon: 1/113 (0.9)	
*NLRP1*	A7	A6	MSI-H (1)	Colon: 1/113 (0.9)	c.1748delA (p.Lys583ArgfsX14)
*NLRP2*	A7	A6	MSI-H (1)	Colon: 1/113 (0.9)	c.3113delA (p.Asn1038ThrfsX4)
*NLRP4*	T7	T6	MSI-H (1)	Gastric: 1/32 (3.1)	c.1496delT (p.Leu499TrpfsX5)

MSI-H: high microsatellite instability.

For the multiregional mutation analysis (4–7 areas), 2 of 16 CRCs analyzed (12.5%) revealed the *NLRP9* frameshift mutations in one region and the wild-type in the other 6 regions (Figure 2). However, we were not able to find histologic differences between the ITH lesions examined by a pathologist. Also, there was no significant difference in patients’ survival nor clinical outcomes between ITH (*n* = 2) and non-ITH (*n* = 14) cases (*p* > 0.05). We analyzed the ITH of *NLRP1, NLRP2* and *NLRP4* frameshift mutation*,* but did not find any ITH of them.

### Immunohistochemistry

**FIGURE 3:**
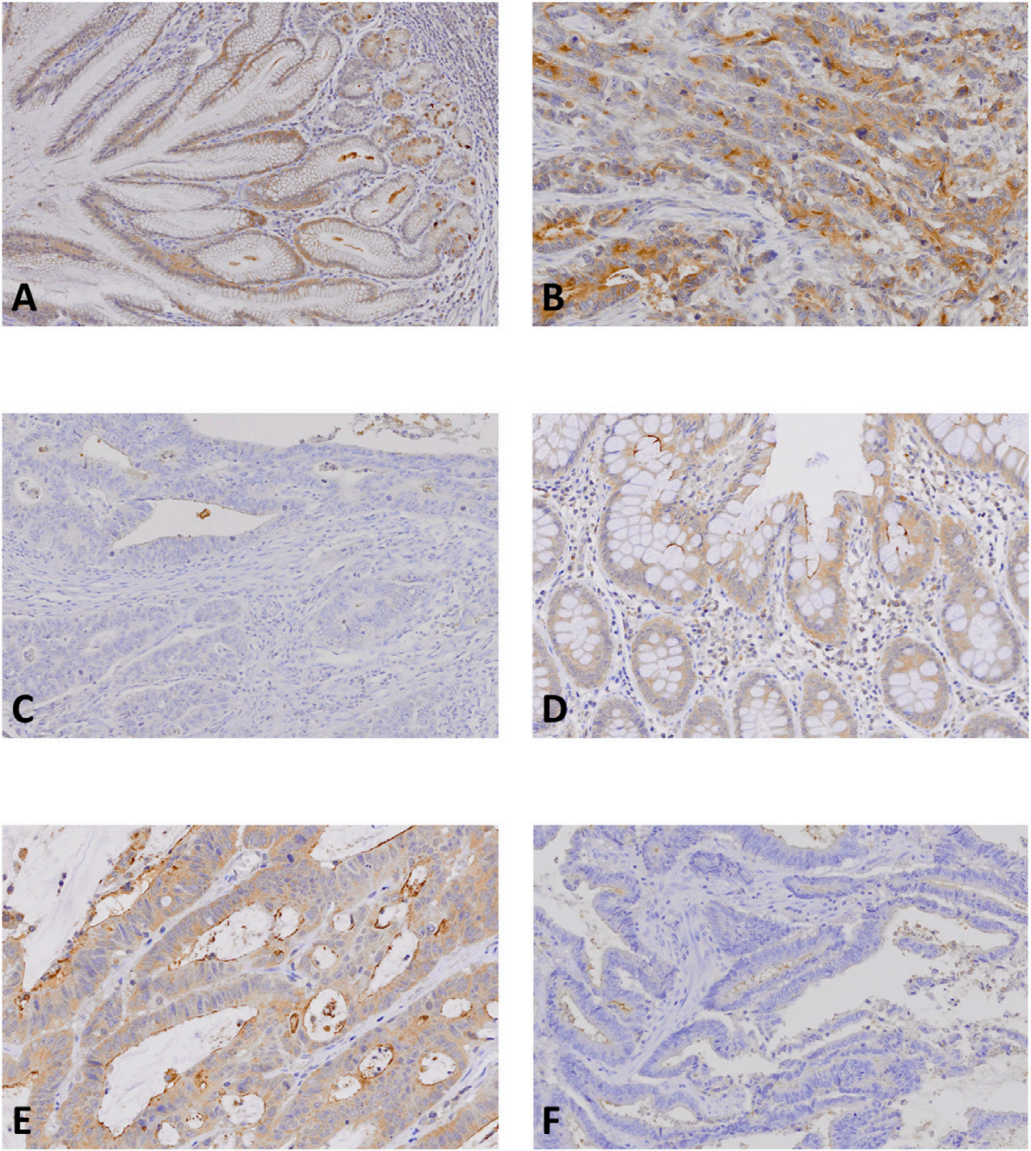
NLRP9 expression in gastric and colon cancer tissues. **(A, D)**: Normal gastric **(A)** and colonic **(D)** mucosal epithelial cells show positive NLRP9 immunostaining. **(B, E)**: Gastric **(B)** and colon **(E)** cancers show positive NLRP9 immunostaining in the cancer cells. **(C, F)**: In gastric **(C)** and colon cancers **(F)** without the *NLRP9* frameshift mutations, the cancer cells show negative NLRP9 immunostaining.

We next studied the expression status of NLRP9 protein since the *NLRP9* mutation was most common among the *NLRP* genes analyzed in this study. In normal gastric and colonic mucosa, NLRP9 was well expressed (IS 6 or 9) by immunohistochemistry (Figures 3A,D). The positive immunostaining was mainly detected in cytosol (Figure 3). The negative control using blocking reagent instead of the primary antibody showed no immunostainings in the tissues. In cancers, the MSS (71.1%, 64/90) and MSI-H (64.8%, 94/145) cancers exhibited no statistically different prevalence in NLRP9 expression (Fisher’s exact test, *p* = 0.197) (Table 3). In both GC and CRC with MSI-H, NLRP9 expression in *NLRP9*-mutated cases was significantly lower than that in *NLRP9*-non-mutated cases (*p* = 0.009 and *p* = 0.016, respectively). There was no significant difference in NLRP9 expression between GC and CRC with MSI-H (*p* > 0.05).

**TABLE 3:** NLRP9 expression in gastric and colon cancers.

	All cases	Positive expression (+, ++)	*p*-value
MSI-H			
Gastric cancer			
Total	32	21 (+: 7, ++:14)	0.009*
With *NLRP9* mutation	4	0 (+:0, ++:0)	
Without *NLRP9* mutation	28	21 (+:7, ++:14)	
Colon cancer			
Total	113	73 (+: 23, ++:50)	0.016*
With *NLRP9* mutation	4	0 (+:0, ++:0)	
Without *NLRP9* mutation	109	72 (+:22, ++:50)	
MSS			
Gastric cancer			
Total	45	32 (+:10, ++:22)	
without *NLRP9* mutation	45	32 (+:10, ++:22)	
Colon cancer			
Total	45	30 (+:7, ++:23)	
without *NLRP9* mutation	45	30 (+:7, ++:23)	

GC: gastric cancer, CRC: coloic cancer, MSI-H: high microsatellite instability, MSS: stable microsatellite instability; * significant.

## Discussion

The role of inflammation is well-known in both development and progression of cancers, and is a hallmark in cancer development [23]. Also, evasion of cell death is known to play an important role in the pathogenesis of cancers [23]. In this sense, the inflammasome associated with both inflammation and cell death could be a good candidate for the cancer research. To find cancer-related alterations in cancer inflammasome, we analyzed frameshift mutations of *NLRP* (*NLRP1, 2, 4* and *9*) genes closely related to the inflammasome in GC and CRC in this study. The mutations were frequent in both MSI-H GC (15.5%) and CRC (5.5%); *NLRP9* mutations revealed ITH; and NLRP9 expression was frequently lost in *NLRP9*-mutated cancers. These data indicate that *NLRPs,* especially *NLRP9,* might be involved in MSI-H cancer pathogenesis. In general, a cancer-related gene may exhibit not only a high incidence of genetic alterations but also functional consequences [23]. For *NLRP9* gene, until now its role has been suggested only in maturation and fertilization of gonocytes [24]. Neither cancer-related functions nor gastrointestinal functions of *NLRP9* gene have been identified. Mouse orthologue Nlrp9b is specifically expressed in intestinal epithelial cells and restricts rotavirus infection by activating pyroptosis [25]. It can be speculated that the *NLRP9* frameshift mutations could inactivate the pyroptosis. However, relationship between pyroptosis and MSI cancers is not known currently. As The Human Protein Atlas database (https://www.proteinatlas.org/) exhibits that NLRP9 expression is evident in normal stomach and colon cells and is strongest in colorectal cancers followed by gastric, pancreatic, testicular and liver cancers, but other cancer tissues are in general weakly stained or negative, suggesting its role in gastrointestinal tract. Together, these data suggest that NLRP9 expression is common in both normal and cancer cells of stomach and colon, and that frameshift mutations and expression loss of *NLRP9* is frequent in GC and CRC with MSI-H. However, it remains to be clarified whether these alterations have a causative role in tumorigenesis, or they simply reflect the phenomenon that MSI-H cancers have an increased frequency of mutations.

In addition to *NLRP9*, we found *NLRP1, 2* and *4* mutations in GC and CRC with MSI-H with lower incidences (Table 2). As mentioned above [9, 13], NLRP1 possesses both oncogenic and TSG activities. Also, NLRP2 revealed TSG functions in glioblastoma and oncogenic functions in bladder cancer [26]. These opposing phenomenon may preclude predicting functional consequences of the frameshift mutations. NLRP4 negatively regulates autophagy processes through an association with beclin1 [27]. There is controversy about the roles of autophagy in cancer (tumor suppressing vs. promoting) [28]. The low incidence as well as the debate suggests that the *NLRP4* mutation may not necessary play an important role in cancer development.

NLRP9 expression was negative in all cancers with its frameshift mutations (Table 3). Anti-NLRP9 antibody adopted in this study was made using a synthetic peptide of amino acids 472-549 (isoform 1). Based on the nucleotide changes of *NLRP9* by the frameshift mutations, 2 amino acid locations (amino acids 7 and 264) would be altered and truncated (Table 2). Parts of pyrin domain (amino acids 1-94), NACHT domain (146-465) and leucine repeat domains (743-907) (www.uniprot.org) would be truncated by the mutations and thus the truncated area may not be detected by the immunohistochemistry.

Our data indicate that the mutational ITH of *NLRP9* is common in the CRC (12.5% of MSI-H CRCs) (Figure 2). ITH is important in driving phenotypic selection and adaptation in response to selective pressures in a cancer [29]. ITH is known to be associated with poor prognosis and clinical outcomes [29]. The ITH in our study could ameliorate the phenotypic consequences of the mutation and further functional and clinical implication of the ITH is required to be defined.

## Data Availability

The raw data supporting the conclusion of this article will be made available by the authors, without undue reservation.
